# Management of Heparin-Induced Thrombocytopenia: A Contemporary Review

**DOI:** 10.3390/jcm13164686

**Published:** 2024-08-09

**Authors:** Jun Yen Ng, Melanie D’Souza, Felanita Hutani, Philip Choi

**Affiliations:** Department of Hematology, Canberra Hospital, Canberra 2605, Australiaphil.choi@act.gov.au (P.C.)

**Keywords:** heparin, thrombocytopenia, thrombosis, antibody-mediated

## Abstract

Heparin-induced thrombocytopenia (HIT) is a life- and limb-threatening immune-mediated emergency classically associated with heparin therapy. This review focuses on type II HIT, characterized by the development of antibodies against platelet-factor 4 (PF4) bound to heparin after exposure, causing life-threatening thrombocytopenia, arterial thrombosis, and/or venous thrombosis. The high morbidity and mortality rates emphasize the need for early recognition and urgent intervention with discontinuation of heparin and initiation of non-heparin anticoagulation. We discuss the management of HIT with an emphasis on recent developments: (i) incorporating the phases of HIT (i.e., suspected, acute, subacute A and B, and remote) into its management, categorized according to platelet count, immunoassay, and functional assay results and (ii) direct-acting oral anticoagulants (DOACs), which are increasingly used in appropriate cases of acute HIT (off-label). In comparison to parenteral options (e.g., bivalirudin and danaparoid), they are easier to administer, are more cost-effective, and obviate the need for transition to an oral anticoagulant after platelet recovery. We also identify the knowledge gaps and suggest areas for future research.

## 1. Introduction

Heparin-induced thrombocytopenia (HIT) is a life- and limb-threatening immune-mediated emergency classically associated with heparin therapy. Although this has been most commonly described with unfractionated heparin (UFH), it has been seen with low-molecular-weight heparin (LMWH), and clinical syndromes with striking similarity in the absence of heparin exposure have been well reported with non-heparin polyanionic substances such as chondroitin sulfate and pentosan polysulfate (PPS) [1]. In a meta-analysis of 15 studies (*n* = 7287), the risks of HIT associated with UFH and LMWH were 2.6% and 0.2%, respectively [2].

HIT is characterized by the development of antibodies against platelet-factor 4 (PF4) bound to heparin after exposure, causing life-threatening thrombocytopenia, arterial thrombosis, and/or venous thrombosis. Various studies have reported high morbidity (60–90%) and mortality rates (6–30%). Even with early recognition and intervention, morbidity and mortality are 7.4% and 1.1% [3,4,5,6,7]. This emphasizes the need for early recognition and urgent intervention with discontinuation of heparin and initiation of non-heparin anticoagulation. In this review, we discuss this antibody-mediated disorder with an emphasis on management, highlighting recent developments. We also review recommendations from societal guidelines, including the American College of Chest Physicians (ACCP) published in 2012, the American Society of Hematology (ASH) published in 2018 (reviewed in 2022), the British Society of Haematology (BSH) published in 2023, the Thrombosis and Haemostasis Society of Australia and New Zealand (THANZ) published in 2019, and Thrombosis Canada published in 2023 [8,9,10,11,12].

## 2. Pathogenesis

HIT is categorized into types I and II. In type I HIT, there is a transient and non-immune thrombocytopenia due to heparin-induced platelet activation and aggregation. This is seen in approximately 10–20% of patients and occurs shortly after heparin exposure (usually 1–4 days). The thrombocytopenia is mild (nadir 100,000/μL), self-limiting without cessation of heparin, and not associated with thrombosis [13,14]. Type I HIT is not further discussed in this review.

**Figure 1:**
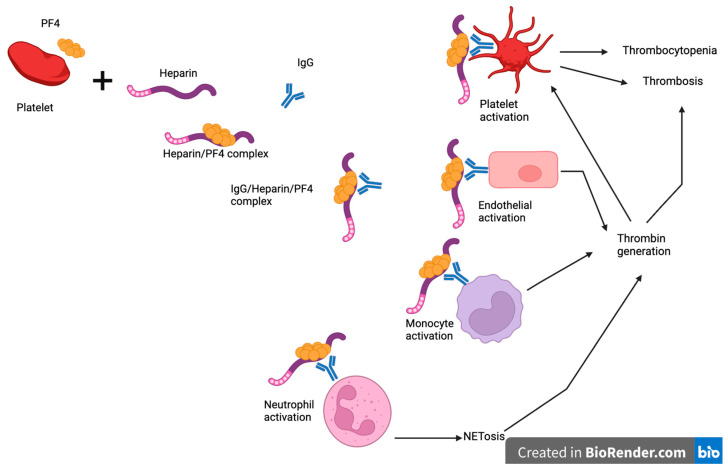
Pathogenesis of heparin-induced thrombocytopenia. Heparin exposure leads to the production of immunoglobulin G (IgG) antibodies against PF4 bound to heparin. These antibodies bind to and activate platelets causing thrombosis and thrombocytopenia. They also bind to endothelium, monocytes, and neutrophils leading to thrombin generation, which promotes thrombosis and further activation of platelets. Created with BioRender.com. PF4: platelet factor 4, IgG: immunoglobulin G.

In type II HIT, heparin exposure leads to the production of immunoglobulin G (IgG) antibodies against PF4 bound to heparin, as illustrated in Figure 1. These antibodies may develop as soon as 4 days post-exposure without an immunoglobulin M (IgM) response suggesting an etiological role for prior sensitization or cross-reactivity [15]. However, it is important to note that despite the detection of anti-PF4/heparin antibody complexes in some patients, they may not be functionally active and not lead to the development of HIT [15].

PF4 is stored in alpha granules and released upon activation of platelets. Upon binding to heparin or other polyanions, PF4 undergoes a conformational change to allow binding with HIT antibodies [16]. These immune complexes bind to platelet FcγRIIA receptors with downstream platelet activation, leading to thrombosis and consumptive thrombocytopenia [15]. More recently, the interaction of the immune complexes with neutrophil FcγRIIA receptors and subsequent NETosis has also been shown to be a major contributing factor in HIT-associated thrombosis by supporting thrombin generation. In addition, HIT antibodies also bind to PF4/glycosaminoglycan complexes on endothelial cells and monocytes with consequent expression of tissue factor and thrombin generation [17]. Thrombin promotes thrombosis and further activation. 

## 3. Diagnosis

In patients with suspected HIT, the “4T” score is commonly used to determine the pretest likelihood of HIT. The score incorporates four clinical and laboratory components: severity of thrombocytopenia, thrombosis or other HIT-associated complications, timing of thrombocytopenia after heparin exposure, and the presence of other causes of thrombocytopenia (Table 1). A total score of 0–3, 4–5, and 6–8 points corresponds to low, intermediate, and high pretest likelihoods, respectively [18].

In patients with low pretest likelihood, further laboratory testing is not recommended except for some patients in the intensive care unit (BSH: Grade 2B), in particular those receiving extracorporeal membrane oxygenation (BSH: Grade 2C) [10]. Another exception is in cases where the pretest probability is uncertain due to missing or unclear data [9,11].

In patients with intermediate and high pretest likelihoods, an immunoassay (e.g., ELISA) is used to detect the presence of HIT antibodies. The test has a rapid turnaround time, can be semi-automated in the case of chemiluminescent platforms, and is more widely available. Functional assays (e.g., serotonin release assay, heparin-induced platelet activation, or heparin-induced platelet aggregometry) are needed to confirm the diagnosis by demonstrating heparin-dependent activation that is abrogated by a high-dose heparin step, which alters the usual stoichiometric relationship between these polyanions and anti-PF4 antibodies needed to trigger platelet activation. Functional assays are generally more time-consuming, require manual handling and expertise, and are generally reserved for major metropolitan tertiary referral centers.

## 4. Management

Once a presumptive diagnosis of HIT is made based on an intermediate to high pretest probability, immediate steps to mitigate the prothrombotic condition must be taken even while waiting for further laboratory testing, including suspension of all heparins and the commencement of a non-heparin anticoagulant.

HIT can be categorized into five phases, starting with suspected cases, where a presumptive clinical diagnosis has been made with pending immunoassay or functional assays. Once confirmed by laboratory testing, cases are categorized as acute HIT until recovery of platelet count (usually defined as ≥150 × 10^9^/L; however, in some studies, the definition also includes the return to baseline if the baseline platelet count was <150 × 10^9^/L, as shown in Table 1). In addition, acute cases without thrombosis are designated as isolated HIT. Subacute phases A and B are characterized by a normal platelet count with positive immunoassays; however, the functional assay is negative in the latter. The remote phase is indicated by seronegativity [19].

**Figure 2:**
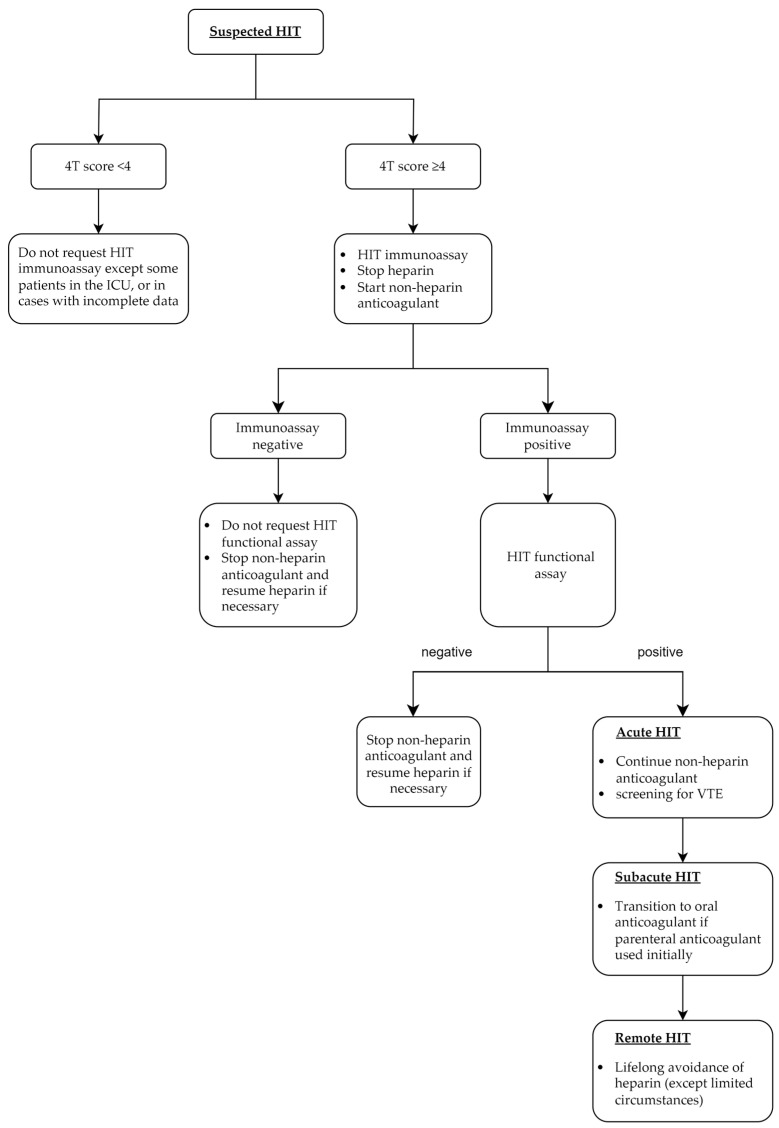
Key management steps in each phase of heparin-induced thrombocytopenia. HIT: heparin-induced thrombocytopenia, ICU: intensive care unit, VTE: venous thromboembolism.

Incorporating these phases into clinical practice may improve communication between healthcare providers and streamline the patient care requirements and management of HIT. For example, as illustrated in Figure 2, the management can be categorized according to the phases of HIT. These are discussed below with a focus on newer data.

### 4.1. Discontinuation of Heparin

All forms of heparin, including UFH and LMWH, need to be discontinued immediately once HIT is suspected with intermediate to high pretest probability [9,10]. Hence, a thorough review is required to evaluate all potential sources, including heparin-coated intravascular catheters, heparin flushes, some prothrombin complex concentrates, peripheral blood stem cells, and total parenteral nutrition products [20]. PPS injections, occasionally used for symptomatic management of osteoarthritis (off-label), also need to be excluded.

### 4.2. Initiation of a Non-Heparin Anticoagulant at Therapeutic Dose

Due to the high risk of venous and/or arterial thrombosis, a non-heparin anticoagulant at therapeutic intensity needs to be commenced immediately once HIT is suspected with intermediate to high pretest probability [9,10]. However, if the bleeding risk is high, ASH suggests initiating a non-heparin anticoagulant at prophylactic intensity [9].

It is important to note that warfarin is not a suitable agent in acute HIT. This is due to the exacerbation of thrombotic risks due to a rapid depletion of protein C and protein S [21]. These anticoagulants are also reduced in the setting of acute thrombosis. For patients already receiving warfarin, reversal with vitamin K is suggested during the acute phase of HIT [8,9]. The use of warfarin after acute HIT is discussed in Section 4.4.2.

Oral and parenteral anticoagulants used in acute HIT are discussed below. Table 2 compares the agents with a dosing and monitoring guide included.

#### 4.2.1. Direct-Acting Oral Anticoagulants (DOACs)

Traditionally, the parenteral non-heparin anticoagulants are the mainstay of therapy in acute HIT. However, there are increasing data and international acceptance for the off-label use of DOACs in suitable patients. In a recent survey of opinions from 102 international experts and practitioners, the majority agreed with the use of rivaroxaban and apixaban in acute HIT (74.5% and 73.5%, respectively), even without initial parenteral anticoagulation (39.2% and 35.3%, respectively) [22].

In addition to the parenteral anticoagulants, ASH suggests DOACs (e.g., rivaroxaban, apixaban, and dabigatran) as suitable alternatives in acute HIT. Specifically, they are suitable for patients who are clinically stable with average bleeding risk [9]. Similarly, Thrombosis Canada recommends DOACs as an option in acute HIT, with a preference for rivaroxaban [11]. In contrast, BSH and THANZ recommend parenteral agents only in acute HIT [10,12].

In comparison to parenteral anticoagulants, the advantages of DOACs include convenience and ease of administration, wide availability, lower risk of bleeding, and no requirement for routine monitoring and titration, which is needed in most parenteral anticoagulants [22]. They can also be continued to the subacute phase, hence avoiding the need to change from parenteral to oral anticoagulants. In addition, a recent pharmacoeconomic analysis favors rivaroxaban (and fondaparinux) over argatroban [23].

Due to their half-life, they may not be suitable for patients needing urgent surgery. They are also not preferred in cases with arterial thrombosis or life-threatening or limb-threatening thromboembolism due to the limited data on DOACs in this setting [9,10]. They are also not suitable for patients with mechanical heart valves, who are pregnant, or those with severe renal and/or liver dysfunction.

More data on rivaroxaban and apixaban have been published on individual DOACs. We summarized the retrospective series and prospective studies reporting the use of DOACs in acute HIT (suspected and confirmed) published in the past 10 years in Table 1.

**Table 2:** Studies reporting outcomes of direct-acting oral anticoagulants in suspected or confirmed acute heparin-induced thrombocytopenia.

Study and Design	Agent and Number of Patients (*n*)	Initial Parenteral Anticoagulation	HIT with Thrombosis	Follow-Up Duration	Thrombosis and Related Outcomes During Follow-Up	Bleeding During Follow-Up	Other Findings or Outcomes During Follow-Up
Davis et al., 2022 [24]	Apixaban (*n* = 51) Rivaroxaban (*n* = 24) Dabigatran (*n* = 2)	63 (81.8%)	38 (49.4%), including 5 arterial thrombosis	3 months from starting DOAC	Thromboembolism, gangrene, or severe limb ischemia requiring amputation: 9 (11.7%); all received initial parenteral anticoagulation, among which 7/9 (77.8%) changed to DOAC when platelet count was <150 × 10^9^/L	Major: 5 (6.5%), CRNMB: 9 (11.7%)	Time to platelet recovery (days): Initial parenteral anticoagulation: 5 (3.25–8) Initial DOAC: 4 (2.5–9.5) All-cause mortality at: 1 month: 1 (1.3%) 3 months: 5 (6.5%) None in patients who received DOAC only (*n* = 14)
Multicenter, retrospective							
Cirbus et al., 2022 [25]	Rivaroxaban (*n* = 7) Apixaban (*n* = 5)	10 (83.3%), 5 (36%) changed to DOAC before platelet recovery	2 (28.5%) in rivaroxaban group, unknown in apixaban group	6 months from discharge	0 in 8 (67%) patients who followed-up	Not reported	
Single center, retrospective							
Albuloushi et al., 2022 [26]	Apixaban (*n* = 21) Rivaroxaban (*n* = 5)	21 (80.8%); 11 (42.3%) changed to DOAC after platelet recovery	16 (61.5%), including 2 arterial thrombosis	1 month	0	0	Time to platelet recovery (days): Initial parenteral anticoagulation (*n* = 18): 5 (2.8–8.3) Initial DOAC (*n* = 5): 6 (2.3–14.5)
Single center, retrospective							
Farasatinasab et al., 2022 [27]	Apixaban (*n* = 30)	0	11 (36.7%)	6 months	0	1 (3.3%): bleeding gastric ulcer at 2 months and apixaban ceased	Time to platelet recovery (days): 5 ± 1.8 Unrelated mortality rate: 16.7%
Single center, prospective, open-label, single-arm							
Wang et al., 2022 [28]	Dabigatran (*n* = 5)	5 (100%), all changed to DOAC before platelet recovery ^	5 (100%), including 3 with arterial embolism	3 months	0	1 (20%) developed gastric bleeding	3 (60%) recovered platelet count at end of follow-up ^ Mortality rate: 1 (20%)
Single center, retrospective							
Carré et al., 2021 [29]	Rivaroxaban (*n* = 6) Apixaban (*n* = 1)	6 (85.7%), 1 (14.3%) changed to DOAC before platelet recovery	1 (14.3%)	12 (4–27.5) months	1 (14.3%), DVT after holding rivaroxaban 5 days pre-surgery;	0	Time to platelet recovery (days) ^: 3 (3–5)
Multicenter, retrospective							
Farasatinasab et al., 2020 [30]	Rivaroxaban (*n* = 42)	0	17 (40.5%)	12 months from starting DOAC	1 (2.3%) developed progressive DVT	0	Time to platelet recovery (days): 4.29 ± 1.78 Unrelated mortality rate: 28.6%
Single center, retrospective							
Nasiripour et al., 2018 [31]	Dabigatran (*n* = 43)	0	Not reported	12 months from starting DOAC	1 (2.3%) developed lower-limb DVT	0	Time to platelet recovery: 7.4 ± 4.3 days (*n* = 41, 95.3%) Unrelated mortality rate: 20%
Single center, retrospective							
Davis et al., 2017 [32]	Apixaban (*n* = 9) Rivaroxaban (*n* = 3)	7 (58.3%)	5 (41.7%), including 1 arterial embolism	19 months	Thromboembolism, gangrene, or critical limb ischemia requiring amputation: 0	Major: 0	Time to platelet recovery (days): mean 7.42 days
Single center, retrospective							
Kunk et al., 2016 [33]	Apixaban (*n* = 10) Rivaroxaban (*n* = 2)	12 (100%), all changed to DOAC after platelet recovery ^	9 (75%), including 1 arterial thrombosis	Median 7 (range 2–39) months	0	Major: 2 (16.7%) **	Time to platelet recovery (days) ^: 1–8
Single center, retrospective							
Warkentin et al., 2016 [34]	Rivaroxaban (*n* = 16)	8 (50%), 2 changed to rivaroxaban when thrombocytopenic #	6 (37.5%)	1 month	Thrombosis or limb amputation: 0	Major: 0	Time to platelet recovery (days) ^†: 7 (4–12) (*n* = 9) Mortality rate 0%
Multicenter, retrospective							
Ong et al., 2016 § [35]	Rivaroxaban (*n* = 9)	0	9 (100%)	Not reported	Thrombosis or limb amputation due to necrosis: 0	0	Time to platelet recovery (days) ^: mean 14, median 8 (range 5–41)
Multicenter, retrospective							
Linkins et al., 2016 [36]	Rivaroxaban (*n* = 12)	7 (58.3%) ‡	6 (50%), including 1 arterial thrombosis	1 month	1 (8.3%, 95% CI, 0.1–37.5%) developed extension of upper arm catheter-associated DVT	1 (8.3%) major bleeding after rivaroxaban held for 9 days	Time to platelet recovery (days) ‡: mean 11, median 7. Unrelated mortality rate: 33.3%
Multicenter, prospective,							
Sharifi et al., 2015 [37]	Dabigatran, rivaroxaban, and apixaban; Total 22 patients.	22 (100%)	7 (31.8%)	19 ± 3 months	Recurrent venous thromboembolism or limb loss: 0	0	Unrelated mortality rate: 27%
Single center, retrospective							

Unless otherwise indicated, data are presented as *n* (%), mean ± standard deviation and median (interquartile range). ^ Platelet recovery is defined as: (i) platelet count ≥150 × 10^9^/L or (ii) if <150 × 10^9^/L at baseline, return to baseline platelet count except Carré et al., Warkentin et al., Ong et al. [29,34,35], which included (i) only; Kunk et al. defined platelet recovery as count of ≥50 × 10^9^/L; not defined in Wang et al. [28]; we applied both (i) and (ii) to report these statistics. See full text for more details. # Platelet count 56 (range 25–107) × 10^9^/L. ** One patient with known gastric varices who was taking clopidogrel developed gastrointestinal bleeding; another patient with locally advanced squamous cell lung cancer developed severe hemoptysis. † One patient never had thrombocytopenia. § Includes 3 cases published by Ng et al. in 2015 [34,38]. ‡ Ten patients had thrombocytopenia at study entry when they commenced or transitioned to rivaroxaban. Nine patients recovered platelet count CI: confidence interval, CRNMB: clinically relevant non-major bleeding, DOAC: direct-acting oral anticoagulants, DVT: deep venous thrombosis, HIT: heparin-induced thrombocytopenia, HITT: heparin-induced thrombocytopenia with thrombosis, IQR: interquartile range.

#### 4.2.2. Danaparoid

Danaparoid inhibits factor Xa and, to a much lesser extent, factor IIa by its effect on antithrombin [39]. It is mainly metabolized by the kidneys, with a half-life elimination of approximately 25 and 2 h for its anti-Xa and anti-IIa activity, respectively. As protamine only partially neutralizes danaparoid, there is no reliable reversal agent. Thus, danaparoid may not be suitable for patients requiring emergency surgery, cardiac surgery, or at high risk of bleeding [9,40]. It is usually administered as a continuous intravenous (IV) infusion after a loading dose bolus. In approximately 10% of cases, danaparoid cross-reactivity is observed with the HIT antibody. In vivo cross-reactivity is rare; however, it is important to consider the possibility in cases with suboptimal platelet response to treatment and/or new thrombotic events [41,42,43]. Danaparoid does not cross the placenta and is suitable for pregnant patients with HIT [10,40,44]. It is also suitable for patients who are breastfeeding [10].

#### 4.2.3. Bivalirudin

Bivalirudin is a direct thrombin inhibitor (DTI). It is metabolized by proteolytic cleavage (80%) and renal excretion (20%) with a short half-life of 25 min [40]. It is administered as a continuous IV infusion. Hence, the agent may be preferred in patients at high risk of bleeding, those who are critically ill, or those who may require urgent surgery. ASH suggests bivalirudin in patients with acute HIT or subacute HIT A who require percutaneous coronary intervention (PCI) over other non-heparin anticoagulants. It is also suggested over UFH in patients with subacute HIT B or remote HIT undergoing PCI [9].

#### 4.2.4. Argatroban

Argatroban is a DTI. It is predominantly metabolized in the liver with a short half-life of 40–50 min [12,19]. Hence, it is not suitable or requires a dose reduction in patients with moderate to severe liver dysfunction (Child–Pugh Class B and C). Due to the short half-life, the agent may be favored in patients who may require emergency surgery or are at high risk of bleeding [9]. It is administered as a continuous IV infusion. For patients with acute HIT or subacute HIT A who require PCI and bivalirudin is not available, the agent may be a suitable alternative [9]. It is an option for pregnant patients with HIT [10].

#### 4.2.5. Fondaparinux

Fondaparinux is a synthetic pentasaccharide that selectively inhibits factor Xa by its effects on antithrombin. It is predominantly excreted by the kidneys, with a long half-life of 17–24 h [19]. It is a subcutaneous injection administered once daily without the need for monitoring. Fondaparinux is an attractive option for clinically stable patients without a high risk of bleeding. Due to the long half-life, the agent is not suitable for cardiac surgery [9]. It is an option for pregnant patients with HIT [10]. However, there is a small amount of in vivo placental transfer; hence, ACCP recommends its use as an alternative if danaparoid is not available [8]. Note that although the risk is low, fondaparinux is associated with autoimmune HIT.

#### 4.2.6. Comparison of Non-Heparin Anticoagulants

The selection of an agent would depend on the availability, cost, clinician experience, and various patient factors, as summarized in Table 3 below, incorporating recent guidelines, reviews, and databases [9,10,12,19,22,40,45,46,47].

### 4.3. Screening for Asymptomatic DVT

Asymptomatic lower-limb DVTs are common in patients with HIT, with an incidence rate as high as 50% reported [48]. In addition, 9.7% of patients who received a central venous catheter (CVC) up to 2 weeks before a diagnosis of HIT were reported to have an upper-limb DVT ipsilateral to the CVC site [49]. Hence, ASH and Thrombosis Canada suggest screening for asymptomatic proximal DVTs in patients with acute isolated HIT using bilateral lower-limb compression ultrasound. In addition, in patients with an upper-limb CVC, ASH also suggests ultrasound of the ipsilateral upper limb [9,11]. Identification of a DVT has significant implications on the duration of anticoagulation, as discussed in Section 4.5.

### 4.4. Transition to Oral Anticoagulant

Platelet count recovery marks the transition to subacute HIT, an important time point to guide the transition to oral and/or outpatient anticoagulation [50]. Other considerations include the need for urgent procedures requiring immediate anticoagulation cessation and safety for oral administration [51].

#### 4.4.1. Direct-Acting Oral Anticoagulants

DOACs offer several advantages over warfarin, particularly without the need for monitoring or titration. ASH suggests DOACs over warfarin in patients with subacute HIT A, with the choice of agent determined by drug/patient factors and clinician experience [9]. BSH supports DOACs in patients with HIT who are clinically stable [10]. Thrombosis Canada recommends either DOACs or warfarin for patients who have recovered their platelet count [11]. THANZ recommends DOACs as an alternative to warfarin for patients who have responded to parenteral anticoagulation [12].

DOACs should be commenced within 2 h of stopping argatroban or bivalirudin, 8–12 h after stopping danaparoid, and 24 h after the last dose of fondaparinux, based on the half-life of each of the drugs, respectively [9,10,52].

The doses of DOACs for subacute HIT are also listed in Table 2.

#### 4.4.2. Warfarin

Patients commencing warfarin in subacute HIT should continue parenteral non-heparin anticoagulation for at least five days and until the target international normalized ratio (INR) is reached [8]. When transitioning from bivalirudin and argatroban, it is important to be aware that these agents also prolong the INR [9].

### 4.5. Duration of Therapeutic Anticoagulation

For HIT patients with thrombosis, three months of anticoagulation is recommended (BSH: Grade 1A) [10]. For HIT patients without thrombosis, ASH suggests anticoagulation until platelet recovery at a minimum [9]. ACCP suggests four weeks of anticoagulation, whereas the BSH recommends anticoagulation until platelet count recovery or at least four weeks, whichever is later [8,10]. Thrombosis Canada recommends at least four weeks of anticoagulation and until platelet recovery [11].

### 4.6. Avoidance of Heparin

Following a diagnosis of HIT, all heparin products should be avoided except those absolutely required, primarily during cardiovascular surgery, coronary angiography, or percutaneous coronary intervention [9,10,11,20]. The diagnosis should be noted in the patient’s medical record, and an alert card or emergency bracelet may be provided to the patient [9,10]. The decision for heparin re-exposure may be informed by evidence of persistent HIT antibodies, positive functional assays, and a careful evaluation of available expertise with alternative anticoagulants (e.g., cardiopulmonary bypass with bivalirudin).

### 4.7. Management during Cardiovascular Intervention

Patients with HIT undergoing cardiovascular surgery or surgeries requiring cardiopulmonary bypass are a challenge due to the need for an intraoperative anticoagulant where heparin is normally the preferred agent in this setting. In general, surgeries should be delayed in patients with HIT until they are negative for the HIT antibody (usually >100 days after diagnosis) [10]. Where this is not possible, recommendations from various societal guidelines pertaining to cardiovascular surgery are discussed below.

ACCP suggests intraoperative bivalirudin over non-heparin anticoagulants or heparin combined with antiplatelets for patients with acute or subacute HIT [8]. ASH suggests one of these options for patients with acute or subacute HIT A depending on the cost, availability, and experience of the treating team: intraoperative bivalirudin, heparin plus a potent antiplatelet (e.g., tirofiban or prostacyclin analogs), or plasma exchange and intraoperative heparin [9]. BSH recommends intraoperative bivalirudin in patients with active or recent HIT with positive antibodies. If bivalirudin is not available and intraoperative heparin is required, plasma exchange with or without intravenous immunoglobulin may be offered [10].

In patients with subacute HIT B or remote HIT, intraoperative anticoagulation with heparin is recommended over non-heparin anticoagulant, plasma exchange and heparin, or heparin combined with an antiplatelet agent. Patients who receive heparin intraoperatively may require platelet count monitoring post-surgery due to the risk of delayed-onset HIT [9]. Otherwise, heparin should always be avoided pre- and post-surgery.

For patients with acute HIT or subacute HIT A who require percutaneous coronary intervention (PCI), bivalirudin is recommended over other non-heparin anticoagulants. Argatroban is an alternative if bivalirudin is not available [9].

### 4.8. Management for Patients Undergoing Renal Replacement Therapy

In acute HIT patients undergoing renal replacement therapy (RRT), a parenteral non-heparin anticoagulant is suggested over heparin and citrate. The options are argatroban, danaparoid, or bivalirudin. After the acute phase, patients requiring ongoing RRT without other indications for anticoagulation can receive citrate or other non-heparin agents to prevent thrombosis of the dialysis circuit [8,9,10].

While DOACs have increasingly become the preferred agents to treat HIT, the optimal dosing regimens and safety in patients with severe renal impairment remain poorly defined.

### 4.9. Summary of Guidelines and Grades of Evidence

Table 4 summarizes the guidelines on management of HIT with grades of evidence included where available.

## 5. Future Directions

We discussed the emerging benefits of delineating HIT into different phases according to the platelet count, immunoassay, and functional assay results. In addition, their use in the research setting may help to reduce heterogeneity in study design, patent recruitment, and streamlining reporting outcomes.

We also reviewed data on the use of DOACs in acute HIT published within the last 10 years. Accumulated evidence is mainly retrospective, with only two prospective studies published to date. Additional prospective data would be helpful, particularly a comparison between DOAC and parenteral-heparin anticoagulants [9]. However, it is important to note challenges reported by previous prospective studies. While heparin is widely used in various clinical settings, HIT is not common. These factors pose difficulties in enrollment and prospective selection of patients [53]. In addition, patients with HIT require timely treatment and are often critically unwell. Hence, thorough consideration of the study methodology, enrollment, and a consent process are required. In addition, the eligibility criteria, such as the threshold for concomitant renal impairment given the frequency in HIT patients, need to be carefully selected [36,53]. Given the increasing acceptance of DOACs for suitable patients with acute HIT and the challenges in designing prospective studies in HIT, establishing an international registry may help facilitate clinician reporting and capturing the real-world outcomes of acute HIT treated with DOACs [9,22].

Better defining of non-canonical HIT presentations remains another important frontier [22]. As we approach consensus definitions of terms such as persistent HIT, refractory HIT, and delayed-onset HIT, we may be better placed to understand the mechanisms of these phenomena, plot their natural history, and design treatment strategies to better target these scenarios.

We have not discussed other PF4-immune diseases in this paper, but we recognize the emerging recognition these disorders have in the field and the future challenges these conditions will bring as we attempt to better identify and treat them [54].

## 6. Conclusions

In this contemporary review on the management of HIT, we discussed the management of HIT with an emphasis on recent developments, namely incorporating the phases of HIT into its management and DOACs for acute HIT.

The delineation of the phases of HIT, incorporating platelet count, immunoassay, and functional assays, may offer several advantages in clinical and research settings. This includes streamlining patient management and care requirements according to the different phases of HIT and facilitating communication between healthcare providers. Additionally, incorporating the phases of HIT in the research setting may help optimize study design and outcome reporting.

There is increasing acceptance of DOACs worldwide in suitable patients with acute HIT, especially clinically stable patients without high risk of bleeding. They offer several advantages over parenteral anticoagulants, such as convenience of use, ease of administration, broad availability, and lower costs. They do not require monitoring or inpatient management and can be continued to the subacute phase of HIT.

Future research on the efficacy of DOACs in HIT would be beneficial, in particular, comparison with parenteral non-heparin anticoagulants. However, the challenges of designing prospective studies in HIT need to be considered. An international registry may capture the real-world outcomes of patients with HIT treated with DOACs.

## Figures and Tables

**Table 1:** Estimating pretest likelihood of heparin-induced thrombocytopenia using the 4T score.

Parameter	0 Points	1 Point	2 Points
Thrombocytopenia	Decrease in platelet count by <30% or nadir <10 × 10^9^/L	Decrease in platelet count by 30–50% or platelet nadir 10–19 × 10^9^/L	Decrease in platelet count by >50% or platelet nadir ≥20 × 10^9^/L
Timing of platelet count decrease in relation to heparin exposure	<4 days without recent exposure	Likely 5–10 days but not definite; after 10 days; or ≤1 day (previous heparin exposure 30–100 days ago)	5–10 days; or ≤1 day (previous heparin exposure within 30 days)
Thrombosis or other sequelae	None	Progressive or recurrent thrombosis; suspected thrombosis; non-necrotizing (erythematous) skin lesions	Confirmed new thrombosis, skin necrosis, acute systemic reaction post intravenous bolus heparin
Other causes of thrombocytopenia	Definite	Possible	None

Adapted from Lo et al., 2006 [18].

**Table 3:** Comparison of non-heparin anticoagulants during acute HIT.

Characteristics	Rivaroxaban	Apixaban	Dabigatran	Danaparoid	Bivalirudin	Argatroban	Fondaparinux
Mechanism of action	Directly inhibits factor Xa	Directly inhibits factor Xa	DTI	Antithrombin-mediated inhibition of factor Xa (mainly) and IIa	DTI	DTI	Antithrombin-mediated inhibition of factor Xa
Clearance	Liver	Liver	Kidney	Kidney	Proteolytic cleavage (80%), kidney (20%)	Liver	Kidney
Half-life	5–9 h	8–15 h	12–17 h	25 h (Xa)	25 min	40–50 min	17–24 h
Route of administration	Oral	Oral	Oral	Continuous IV infusion	Continuous IV infusion	Continuous IV infusion	SC injection
Dosing	HIT with thrombosis: 15 mg twice daily for 3 weeks, then 20 mg once daily Isolated HIT: 15 mg twice daily until platelet count recovery	HIT with thrombosis: 10 mg twice daily for 1 week, then 5 mg twice daily Isolated HIT: 5 mg twice daily until platelet count recovery	HIT with thrombosis: 150 mg twice daily (requires 5 days or more of parenteral anticoagulation initially) Isolated HIT: 150 mg twice daily until platelet count recovery	Bolus: <60 kg: 1500 units 60–75 kg: 2250 units 75–90 kg: 3000 units >90 kg: 3750 units Accelerated initial infusion: 400 units/hour for 4 h, then 300 units/hour for 4 h Maintenance infusion: Normal renal function: 200 units/hour Renal dysfunction: 150 units/hour	Bolus: none Maintenance: Normal organ function: 0.15 mg/kg/h Renal or liver dysfunction: dose reduction may be required	Bolus: none Maintenance: Normal organ function: 2 µg/kg/min Hepatic dysfunction (bilirubin > 1.5 mg/dl): 0.5–1.2 µg/kg/min Anasarca, heart failure, post-cardiac surgery: 0.5–1.2 µg/kg/min	<50 kg: 5 mg daily 50–100 kg: 7.5 mg daily >100 kg: 10 mg daily CrCl 30–50 mL/min: use with caution
Monitoring	Not routinely required	Not routinely required	Not routinely required	Anti-Xa (danaparoid-specific): 0.5–0.8 u/ml	APTT: 1.5 to 2.5 times of baseline	APTT: 1.5 to 3 times of baseline value	Not routinely required
Preferred settings	Clinically stable without high risk of bleeding	Clinically stable without high risk of bleeding	Clinically stable without high risk of bleeding	Liver dysfunction Pregnancy Hemodialysis	PCI Hemodialysis Liver dysfunction	PCI CrCl < 30 mL/min Hemodialysis Pregnancy	Clinically stable without high risk of bleeding Pregnancy
Precautions and/or unsuitable settings	Pregnancy Life-threatening or limb-threatening thrombosis Child–Pugh class B and C liver dysfunction Arterial thrombosis Mechanical heart valves	Pregnancy Life-threatening or limb-threatening thrombosis Child–Pugh class C liver dysfunction preceding therapy Arterial thrombosis Mechanical heart valves	Pregnancy Life-threatening or limb-threatening thrombosis CrCl ≤ 30 mL/min Arterial thrombosis Mechanical heart valves	In vivo cross-reactivity with HIT antibodies is rare but possible [43] Urgent surgery Cardiac surgery High risk of bleeding	Prolongs PT; caution if transitioning to warfarin High cost	Prolongs PT; caution if transitioning to warfarin Child–Pugh class B and C liver dysfunction: avoid or dose-reduce High cost	Associated with autoimmune HIT Urgent surgery Cardiac surgery High risk of bleeding CrCl < 30 mL/min: avoid use

APTT: activated partial thromboplastin time, CrCl: creatinine clearance, DTI: direct thrombin inhibitor, HIT: heparin-induced thrombocytopenia, IV: intravenous, PCI: percutaneous coronary intervention, PT: prothrombin time, SC: subcutaneous. This table is intended as a guide only. Selecting an anticoagulant in HIT is a complex decision requiring careful clinical judgment.

**Table 4:** Summary of guidelines on management of HIT.

Guidelines	Recommendations and Grade of Evidence (Where Available)
Discontinuation of heparin	
ASH, 2018 [9]	Recommended in patients with intermediate to high probability of HIT (strong recommendation; moderate certainty in the evidence about effects).
BSH, 2023 [10]	Recommended in patients with intermediate to high probability of HIT (Grade 1C).
THANZ, 2019 [12]	Recommended in patients with intermediate to high probability of HIT and a positive immunoassay result (Grade 1C).
Thrombosis Canada [11]	Recommended in patients with intermediate to high probability of HIT.
Initiation of a non-heparin anticoagulant at therapeutic dose	
ASH, 2018 [9]	Recommended in patients with intermediate to high probability of HIT (strong recommendation; moderate certainty in the evidence about effects) except in patients with high bleeding risk, where prophylactic anticoagulation is suggested (conditional recommendation; moderate certainty in the evidence about effects).
BSH, 2023 [10]	Recommended in patients with intermediate to high probability of HIT (Grade 1C).
THANZ, 2019 [12]	Recommended in patients with intermediate to high probability of HIT and a positive immunoassay result (Grade 1C).
Thrombosis Canada [11]	Recommended in patients with intermediate to high probability of HIT.
Reversal of warfarin	
ACCP, 2012 [8]	Suggested in patients diagnosed with HIT (Grade 2C).
ASH, 2018 [9]	Recommended in patients with acute HIT (strong recommendation, moderate certainty in the evidence about effects).
Thrombosis Canada [11]	Recommended in patients diagnosed with HIT.
Direct-acting oral anticoagulants	
ASH, 2018 [9]	Suggested as suitable alternatives to parenteral agents in acute HIT (conditional recommendation; very low certainty in the evidence about effects). Suggested over VKA in patients with subacute HIT A, with the choice of agent determined by drug/patient factors and clinician experience. DOACs are preferred in clinically stable patients without a high risk of bleeding (conditional recommendation, moderate certainty in the evidence about effects).
BSH, 2023 [10]	Suggested in patients with clinically stable HIT (Grade 2C).
Thrombosis Canada [11]	Recommended as an option in acute HIT, with a preference for rivaroxaban. DOACs or warfarin can be used for long-term anticoagulation after platelet count recovery.
THANZ, 2019 [12]	An alternative to warfarin after patients have responded to parenteral anticoagulants (Grade 2C).
Danaparoid	
ACCP, 2012 [8]	Suitable for pregnant patients with acute or subacute HIT (Grade 2C).
BSH, 2023 [10]	Suitable for pregnant patients with HIT (Grade 2C).
Thrombosis Canada [11]	An option for pregnant patients with HIT.
Argatroban	
BSH, 2023 [10]	Suitable for pregnant patients with HIT (Grade 2C).
THANZ, 2019 [12]	Preferred anticoagulant in patients with severe renal impairment (creatinine clearance < 30 mL/min) (Grade 2C).
Fondaparinux	
ACCP, 2012 [8]	An option in pregnant patients with HIT if danaparoid is not available (Grade 2C).
BSH, 2023 [10]	Suitable for pregnant patients with HIT (Grade 2C).
Thrombosis Canada [11]	An alternative for pregnant patients with HIT if danaparoid is not available.
Screening for asymptomatic VTE	
ASH, 2018 [9]	Bilateral lower-extremity compression ultrasound is suggested in patients with acute isolated HIT (conditional recommendations; very low certainty in the evidence about effects). Ultrasound of the ipsilateral upper limb is suggested in patients with acute isolated HIT with an upper-limb central venous catheter (conditional recommendations; very low certainty in the evidence about effects).
Thrombosis Canada [11]	Suggested especially in the presence of additional risk factors for VTE.
Warfarin	
ACCP, 2012 [8]	Patients commencing VKA in subacute HIT should continue parenteral non-heparin anticoagulation for at least five days and until the target INR is reached (Grade 2C).
BSH, 2023 [10]	Recommended with appropriate bridging with a parenteral non-heparin anticoagulant once platelet count has normalized or returned to baseline (Grade 1A).
Thrombosis Canada [11]	DOACs or warfarin can be used for long-term anticoagulation after platelet count recovery. Overlap with a HIT-safe anticoagulant at therapeutic dose for ≥five days and until the INR is therapeutic.
Duration of therapeutic anticoagulation	
ACCP, 2012 [8]	For patients with thrombosis, three months of anticoagulation is suggested. For patients without thrombosis, anticoagulate for four weeks.
ASH, 2018 [9]	For patients without thrombosis, anticoagulate until platelet recovery at minimum (conditional recommendation; very low certainty in the evidence). The panel suggests against anticoagulation for ≥three months unless there is persisting HIT without platelet count recovery (conditional recommendations; very low certainty in the evidence).
BSH, 2023 [10]	For patients with thrombosis, three months of anticoagulation is recommended (Grade 1A). For patients without thrombosis, anticoagulate until platelet count recovery or at least 4 weeks, whichever is later (Grade 1B).
Thrombosis Canada [11]	At least four weeks of anticoagulation and until platelet recovery to baseline.
Avoidance of heparin	
ACCP, 2012 [8]	Fondaparinux at full therapeutic doses is suggested in patients with a past history of HIT who have acute thrombosis (non-HIT related) until the transition to VKA can be achieved (Grade 2C).
ASH, 2018 [9]	A non-heparin anticoagulant is recommended in patients with remote HIT who require treatment or prophylaxis against VTE (strong recommendation; very low certainty in the evidence about effects).
BSH, 2023 [10]	Re-exposure to heparin should be avoided unless essential for patients with a history of HIT (Grade 1A).
Thrombosis Canada [11]	For patients with previous HIT, heparin or LMWH should not be given without discussing it with a specialist.
Management during cardiac surgery	
ACCP, 2012 [8]	For patients with acute HIT requiring nonurgent cardiac surgery, it is recommended to delay the surgery (if possible) until resolution of HIT and the HIT antibodies are negative (Grade 2C). For patients with acute or subacute HIT, intraoperative bivalirudin is suggested over non-heparin anticoagulants or heparin combined with antiplatelets (Grade 2C). For patients with previous HIT without persisting HIT antibodies, short-term heparin is suggested over non-heparin anticoagulants (Grade 2C). For patients with previous HIT and persisting HIT antibodies, non-heparin anticoagulants are suggested over heparin or LMWH (Grade 2C).
ASH, 2018 [9]	For patients with acute or subacute HIT A, one of these options is suggested depending on the cost, availability, and experience of the treating team: intraoperative bivalirudin, heparin plus a potent antiplatelet (e.g., tirofiban or prostacyclin analogs), or plasma exchange and intraoperative heparin (conditional recommendation; low certainty in the evidence about effects). For patients with subacute HIT B or remote HIT, intraoperative anticoagulation with heparin is recommended over non-heparin anticoagulant, plasma exchange and heparin, or heparin combined with an antiplatelet agent (conditional recommendation; very low certainty in the evidence about effects).
BSH, 2023 [10]	For patients with active or recent HIT with positive HIT antibodies, bivalirudin is recommended if surgery cannot be delayed (Grade 2B). If bivalirudin is unavailable and intraoperative heparin is required, plasma exchange with or without intravenous immunoglobulin may be offered (Grade 2C).
Management during percutaneous coronary intervention	
ACCP, 2012 [8]	For patients with acute HIT or subacute HIT, bivalirudin (Grade 2B) or argatroban (Grade 2C) is suggested over other non-heparin anticoagulants. For patients with previous HIT without persisting HIT antibodies, the recommendation is the same as above.
ASH, 2018 [9]	For patients with acute or subacute HIT A, bivalirudin is suggested over other non-heparin anticoagulants (conditional recommendation; low certainty in the evidence). Argatroban might be a suitable alternative if bivalirudin is unavailable or a lack of institutional experience. For patients with subacute HIT B or remote HIT, bivalirudin is suggested over heparin (conditional recommendation; very low certainty in the evidence).
BSH, 2023 [10]	For patients with active or recent HIT with positive HIT antibodies, bivalirudin is recommended if the procedure cannot be delayed (Grade 2B).
Management during renal replacement therapy	
ACCP, 2012 [8]	For patients with acute or subacute HIT, argatroban or danaparoid is suggested over other non-heparin anticoagulants (Grade 2C). For patients with previous HIT requiring ongoing RRT or catheter locking, regional citrate is suggested over heparin or LMWH (Grade 2C).
ASH, 2018 [9]	For patients with acute HIT receiving RRT and requiring anticoagulation to prevent thrombosis of the dialysis circuitry, argatroban, danaparoid, or bivalirudin is suggested over other non-heparin anticoagulants (conditional recommendation; very low certainty in the evidence about effects). For patients with acute HIT receiving RRT and requiring anticoagulation to prevent thrombosis of the dialysis circuitry only, regional citrate is suggested over heparin and other non-heparin anticoagulants (conditional recommendation; very low certainty in the evidence about effects).
BSH, 2023 [10]	For patients with active HIT, a non-heparin anti-coagulant such as argatroban or danaparoid should be given rather than citrate anti-coagulation (Grade 2B). For patients with previous HIT, a non-heparin anti-coagulant such as argatroban, danaparoid, or citrate is suggested (Grade 1C).

ACCP: American College of Chest Physicians, ASH: American Society of Hematology, BSH: British Society of Haematology, DOACs: direct-acting oral anticoagulants, HIT: heparin-induced thrombocytopenia, INR: international normalized ratio, LMWH: low-molecular-weight heparin, RRT: renal replacement therapy, THANZ: Thrombosis and Haemostasis Society of Australia and New Zealand, VKA: vitamin K antagonist, VTE: venous thromboembolism.

## Data Availability

No new data were created or analyzed in this study. Data sharing is not applicable to this article.
